# Myeloperoxidase and its derivative hypochlorous acid combined clinical indicators predict new-onset atrial fibrillation in sepsis: a case-control study

**DOI:** 10.1186/s12872-024-04034-3

**Published:** 2024-07-19

**Authors:** Hui Dai, Jiawei Ye, Shangyuan Wang, Xingyao Li, Wenjie Li

**Affiliations:** Department of Emergency, Xinhua Hospital, Shanghai Jiao Tong University School of Medicine, Shanghai, 200092 China

**Keywords:** Sepsis, New-onset atrial fibrillation, Myeloperoxidase, Hypochlorous acid, Nomogram model

## Abstract

**Backgroud:**

New-onset atrial fibrillation (NOAF) is a common complication of sepsis and linked to higher death rates in affected patients. The lack of effective predictive tools hampers early risk assessment for the development of NOAF. This study aims to develop practical and effective predictive tools for identifying the risk of NOAF.

**Methods:**

This case-control study retrospectively analyzed patients with sepsis admitted to the emergency department of Xinhua Hospital, Shanghai Jiao Tong University School of Medicine from September 2017 to January 2023. Based on electrocardiographic reports and electrocardiogram monitoring records, patients were categorized into NOAF and non-NOAF groups. Laboratory tests, including myeloperoxidase (MPO) and hypochlorous acid (HOCl), were collected, along with demographic data and comorbidities. Least absolute shrinkage and selection operator regression and multivariate logistic regression analyses were employed to identify predictors. The area under the curve (AUC) was used to evaluate the predictive model’s performance in identifying NOAF.

**Results:**

A total of 389 patients with sepsis were included in the study, of which 63 developed NOAF. MPO and HOCl levels were significantly higher in the NOAF group compared to the non-NOAF group. Multivariate logistic regression analysis identified MPO, HOCl, tumor necrosis factor-α (TNF-α), white blood cells (WBC), and the Acute Physiology and Chronic Health Evaluation II (APACHE II) score as independent risk factors for NOAF in sepsis. Additionally, a nomogram model developed using these independent risk factors achieved an AUC of 0.897.

**Conclusion:**

The combination of MPO and its derivative HOCl with clinical indicators improves the prediction of NOAF in sepsis. The nomogram model can serve as a practical predictive tool for the early identification of NOAF in patients with sepsis.

## Background

Sepsis is a life-threatening organ dysfunction caused by a dysregulated host response to infection [1]. Patients with sepsis exhibit a higher incidence of atrial fibrillation (AF) compared to those without sepsis. A meta-analysis concluded that pooled incidence was 189 hospitalized sepsis cases per 100,000 individuals-years and a mortality rate of 26.7% [2]. The incidence of new-onset atrial fibrillation (NOAF) in septic patients is estimated to range between 10% and 46% [3]. NOAF in patients with sepsis typically indicates a poor prognosis, resulting in significantly longer hospital stays and an increased risk of long-term stroke and death [4, 5]. Given the risk of acute hemodynamic compromise and the poor long-term prognosis associated with NOAF during sepsis, early identification of modifiable risk factors is crucial for guiding the prevention and treatment of NOAF in sepsis.

Inflammation and immunity are known to play a causal role in the occurrence and development of AF [6–8]. Several inflammatory cytokines and T-cell surface antigens, including C-reactive protein (CRP), uric acid/albumin ratio, interleukin (IL)-6, IL-1β, myeloperoxidase (MPO), tumor necrosis factor-α (TNF-α), cluster of differentiation (CD)3, CD4, and CD8, have been identified as biomarkers for predicting the incidence of AF and/or the outcome of AF ablation [9–14]. MPO is a heme protease with a heme cofactor and is a member of the heme peroxidase superfamily. Its primary function is to catalyze the formation of hypochlorous acid (HOCl) from chloride ions, form free radicals with oxidizing ability, and kill bacteria and invasive pathogenic microorganisms. Besides its role in defense, MPO is also implicated in the pathogenesis of various cardiovascular diseases [15]. Studies show that MPO can be involved in the atherogenic process through several mechanisms and can also promote ventricular remodeling due to ischemic arrhythmias [16–18]. Peripheral blood MPO levels are identified as an independent predictor of prognosis in patients with acute myocardial infarction [19]. MPO can also catalyze the conversion of matrix metalloproteinase precursors to activated matrix metalloproteinases, which regulate extracellular matrix degradation, cause atrial fibrosis, and promote the development of AF [20]. However, the potential of MPO and HOCl as predictors of NOAF in sepsis remains unclear.

To address this issue, we conducted a retrospective study to analyze the predictive role of MPO and HOCl for NOAF in sepsis.

## Methods

### Study population

**Fig. 1:**
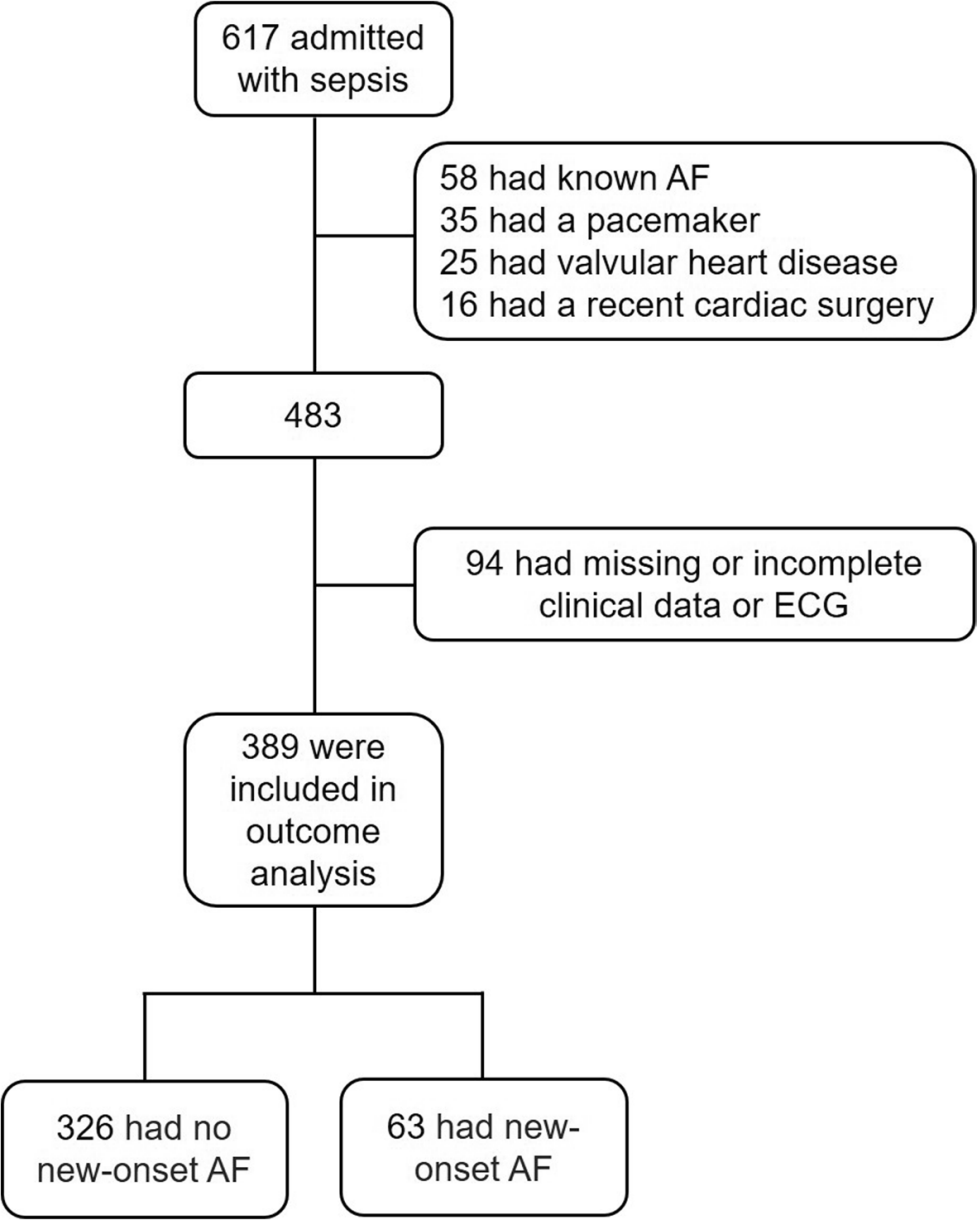
The flowchart of patient inclusion. AF: atrial fibrillation; ECG: electrocardiogram

The study was a single-center, case-control study that enrolled patients with sepsis who presented to the emergency department of Xinhua Hospital, Shanghai Jiao Tong University School of Medicine from September 2017 to January 2023. The diagnostic criteria for sepsis were based on the 2016 joint release of sepsis version 3.0 by the American Society of Critical Care Medicine (SCCM) and the European Society of Intensive Care Medicine (ESICM) [1]. The criteria included: (i) patients with confirmed or suspected infection, and (ii) a Sequential Organ Failure Assessment (SOFA) score of ≥ 2. Patients with sepsis were observed for the occurrence of NOAF within 7 days after hospital admission. The identification of NOAF in sepsis was based on electrocardiographic reports and electrocardiogram (ECG) monitoring records. NOAF in sepsis was defined as: (i) no history of AF, and (ii) absence of P-waves and irregular ventricular activity lasting more than 30 s. The exclusion criteria were: (i) previous history of AF, (ii) recent history of cardiac surgery, (iii) pacemaker implantation, (iv) valvular heart disease, and (v) missing or incomplete clinical data or electrocardiograms. Figure 1 presents the recruitment flowchart.

### Data collection

Baseline characteristics, including demographic data, laboratory parameters, and comorbidities, were collected by reviewing medical records. The following data were obtained within 24 h of patient admission: age, sex, comorbidities, site of infection, white blood cells (WBC), neutrophils, neutrophil percentage, CRP, procalcitonin (PCT), TNF-α, MPO, HOCl, alanine aminotransferase (ALT), aspartate aminotransferase (AST), creatinine (CR), troponin I (TNI), N-terminal pro-brain natriuretic peptide (NT-proBNP), potassium, triglyceride (TG), total cholesterol (TC), high-density lipoprotein cholesterol (HDL-c), low-density lipoprotein cholesterol (LDL-c), CD3, CD4, CD8, CD3%, CD4%, CD8%, and CD4/CD8 ratio. Parameters from transthoracic echocardiography included: left atrium diameter (LAD), left ventricular end-diastolic dimension (LVEDD), left ventricular end-systolic dimension (LVESD), left ventricular ejection fraction (LVEF), and left ventricular fractional shortening (LVFS). Scores from severity of illness classification systems, including the SOFA score and the Acute Physiology and Chronic Health Evaluation II (APACHE II) score, were recorded for each individual at the time of admission. mRNA sequencing and detection of MPO and HOCl levels using the patients’ previous blood specimens. The study was approved by the Ethics Committee of Xinhua Hospital Affiliated to Shanghai Jiao Tong University School of Medicine (no. XHEC-D-2023-079). Each patient signed an informed consent form.

### mRNA sequencing

Total RNA from patients was extracted according to the instructions of the PAXgene Blood miRNA Kit (Qiagen, Germany). This was followed by sequencing, data analysis, quality control, mapping of reads to the reference genome, prediction of novel transcripts, gene functional annotation, quantification of gene expression levels, and differential expression analysis. The relevant data can be accessed through PRJNA953162.

### Detection of MPO and HOCl

According to the kit instructions (MPO: WELLBI, China; HOCl: AAT Bioquest, USA), serum MPO levels were measured by colorimetry, and serum HOCl levels were measured by fluorescence colorimetry.

### Statistical analysis

Statistical analyses were performed using SPSS version 26 and R version 4.1.3, with differences considered statistically significant when the *P* value was < 0.05. Continuous variables were presented as medians and interquartile ranges, while categorical variables were expressed as frequencies and percentages. The Wilcoxon rank-sum test was used to compare continuous variables between the two groups, and the Fisher exact test was used for categorical variables. Least absolute shrinkage and selection operator (LASSO) regression was employed to screen variables and select those with non-zero coefficients. Multivariate logistic regression analysis was used to assess the association between risk variables and NOAF.

## Results

### Study population and incidence of NOAF

A total of 617 patients with sepsis were included in this study. The incidence of NOAF within 7 days of admission was evaluated in 389 eligible patients based on the inclusion and exclusion criteria. Of these, 326 patients did not develop NOAF, while 63 patients did, resulting in an incidence of 16.2% for NOAF within 7 days of admission (Fig. 1).

### Characteristics of septic patients with and without NOAF

The characteristics of septic patients, grouped by their AF status, were compared in Table 1. Patients in the NOAF group were significantly older than those in the non-NOAF group (median: 76 vs. 71 years, *P* = 0.003). In terms of comorbidities, patients with NOAF had a higher incidence of coronary artery disease (CAD) and heart failure compared to those without NOAF. However, there were no significant differences between the two groups in hypertension, diabetes mellitus (DM), chronic obstructive pulmonary disease (COPD), stroke, malignancy, and renal insufficiency. There was also no statistically significant difference regarding the site of infection.

**Table 1:** Characteristics of septic patients with and without NOAF

Characteristics	Overall patients (*n* = 389)		*P* value
	non-NOAF (*n* = 326)	NOAF (*n* = 63)	
Age, years	71 (64–81)	76 (69–83)	0.003
Male, n (%)	206 (63.19)	36 (57.14)	0.365
Comorbidities, n (%)			
Hypertension	174 (53.37)	40 (63.49)	0.139
CAD	83 (25.46)	27 (42.86)	0.005
Heart failure	35 (10.74)	13 (20.63)	0.029
DM	123 (37.73)	25 (39.68)	0.770
COPD	22 (6.75)	6 (9.52)	0.607
Stroke	40 (12.27)	11 (17.46)	0.264
Malignancy	6 (1.84)	4 (6.35)	0.102
Renal insufficiency	29 (8.90)	7 (11.11)	0.579
Infection site, n (%)			
Respiratory tract	154 (47.24)	34 (53.97)	0.328
Gastrointestinal tract	80 (24.54)	10 (15.87)	0.135
Urinary tract	84 (25.77)	17 (26.98)	0.840
Skin and soft tissue	6 (1.84)	1 (1.59)	1.000
Nervous system	2 (0.61)	1 (1.59)	0.982
Laboratory			
WBC (10^9^/L)	9.1 (6.46–13.32)	14.4 (10.6-19.64)	< 0.001
Neutrophils (10^9^/L)	7.5 (4.83–11.8)	11.67 (8.85–17.73)	< 0.001
Neutrophil percentage (%)	83.55 (76-89.6)	88.8 (85.2–92.1)	< 0.001
CRP (mg/L)	122 (52–160)	160 (91–160)	0.005
PCT (ng/mL)	1.28 (0.22–11.1)	12.53 (0.75–58.3)	< 0.001
TNF-α (pg/mL)	18.2 (12.45–29.38)	33.4 (22.6–55.7)	< 0.001
CD3%	66 (56.7-72.97)	66.35 (57.26–74.8)	0.655
CD4%	40.17 (32.13–48.47)	38.4 (30.75–46.32)	0.414
CD8%	21.38 (15.92–28.86)	25.16 (17.58–31.92)	0.109
CD4/CD8 ratio	1.84 (1.19–2.78)	1.6 (1.11–2.24)	0.115
CD3 (cells/μL)	548.85 (336.44–794.80)	430.19 (332.72-664.85)	0.143
CD4 (cells/μL)	336.65 (207.65-500.04)	255.7 (181.85-429.65)	0.060
CD8 (cells/μL)	178.03 (110.5-288.43)	182.61 (102.69-242.57)	0.798
ALT (U/L)	25 (16-46.25)	25 (14–42)	0.569
AST (U/L)	35 (23–59)	39 (24–55)	0.382
CR (umol/L)	78 (60–114)	114.7 (76.30-171.60)	< 0.001
TNI (ng/mL)	0.03 (0.01–0.09)	0.07 (0.03–0.47)	< 0.001
NT-proBNP (pg/mL)	740.9 (242.68–2509)	2828 (1231–7270)	< 0.001
Potassium (mmol/L)	3.66 (3.42-4)	3.83 (3.37–4.22)	0.136
TG (mmol/L)	1.3 (0.89–1.75)	1.16 (0.92–1.67)	0.390
TC (mmol/L)	3.49 (2.78–4.07)	3.27 (2.59–4.07)	0.253
HDL-c (mmol/L)	0.86 (0.67–1.09)	0.82 (0.55–1.11)	0.474
LDL-c (mmol/L)	1.84 (1.24–2.43)	1.51 (1.05–1.99)	0.005
Echocardiography			
LVEF (%)	66.1 (64-68.20)	65 (62–68)	0.028
LVFS (%)	37 (35–39)	35.8 (32–38)	0.005
LVEDD (mm)	48.4 (46-51.20)	48 (46.20–51)	0.845
LVESD (mm)	31 (29.28-33)	31.3 (30–34)	0.101
LAD (mm)	36.65 (33.28-39)	35 (32.90–37.80)	0.116
Severity on admission			
SOFA score	2 (2–4)	4 (3–5)	< 0.001
APACHE II score	10 (8–13)	14 (10–18)	< 0.001

*ALT *alanine aminotransferase, *APACHE II *Acute Physiology and Chronic Health Evaluation II, *AST *aspartate aminotransferase, *CAD *coronary artery disease, *CD *cluster of differentiation, *COPD *chronic obstructive pulmonary disease, *CR *creatinine *CRP *C-reactive protein, *DM *diabetes mellitus, *HDL-c *high-density lipoprotein cholesterol, *LAD *left atrium diameter, *LDL-c *low-density lipoprotein cholesterol, *LVEDD *left ventricular end-diastolic dimension, *LVEF *left ventricular ejection fraction *LVESD *left ventricular end-systolic dimension, *LVFS *left ventricular fractional shortening, *NOAF *new-onset atrial fibrillation, *NT-proBNP *N-terminal pro-brain natriuretic peptide, *PCT *procalcitonin, *SOFA *Sequential Organ Failure Assessment, *TC *total cholesterol, *TG *triglyceride, *TNF-α *tumor necrosis factor-α, *TNI *troponin I, *WBC *white blood cells

Statistical differences were observed between the two groups in WBC, neutrophils, neutrophil percentage, CRP, PCT, TNF-α, CR, TNI, NT-proBNP, and LDL-c. However, no statistical differences were noted in ALT, AST, potassium, TG, TC, HDL-c, CD3, CD4, CD8, CD3%, CD4%, CD8%, and CD4/CD8 ratio.

On echocardiography, LVEF and LVFS were lower in the NOAF group than in the non-NOAF group, whereas LVEDD, LVESD, and LAD were not statistically different between the two groups. Patients with NOAF had significantly higher SOFA and APACHE II scores, which are widely used to evaluate the severity of septic patients. There were statistically significant differences in SOFA and APACHE II scores between the non-NOAF and NOAF groups, with SOFA and APACHE II scores of 2 vs. 4 and 10 vs. 14, respectively.

### Serum sequencing analysis and detection of MPO and HOCl levels

**Fig. 2:**
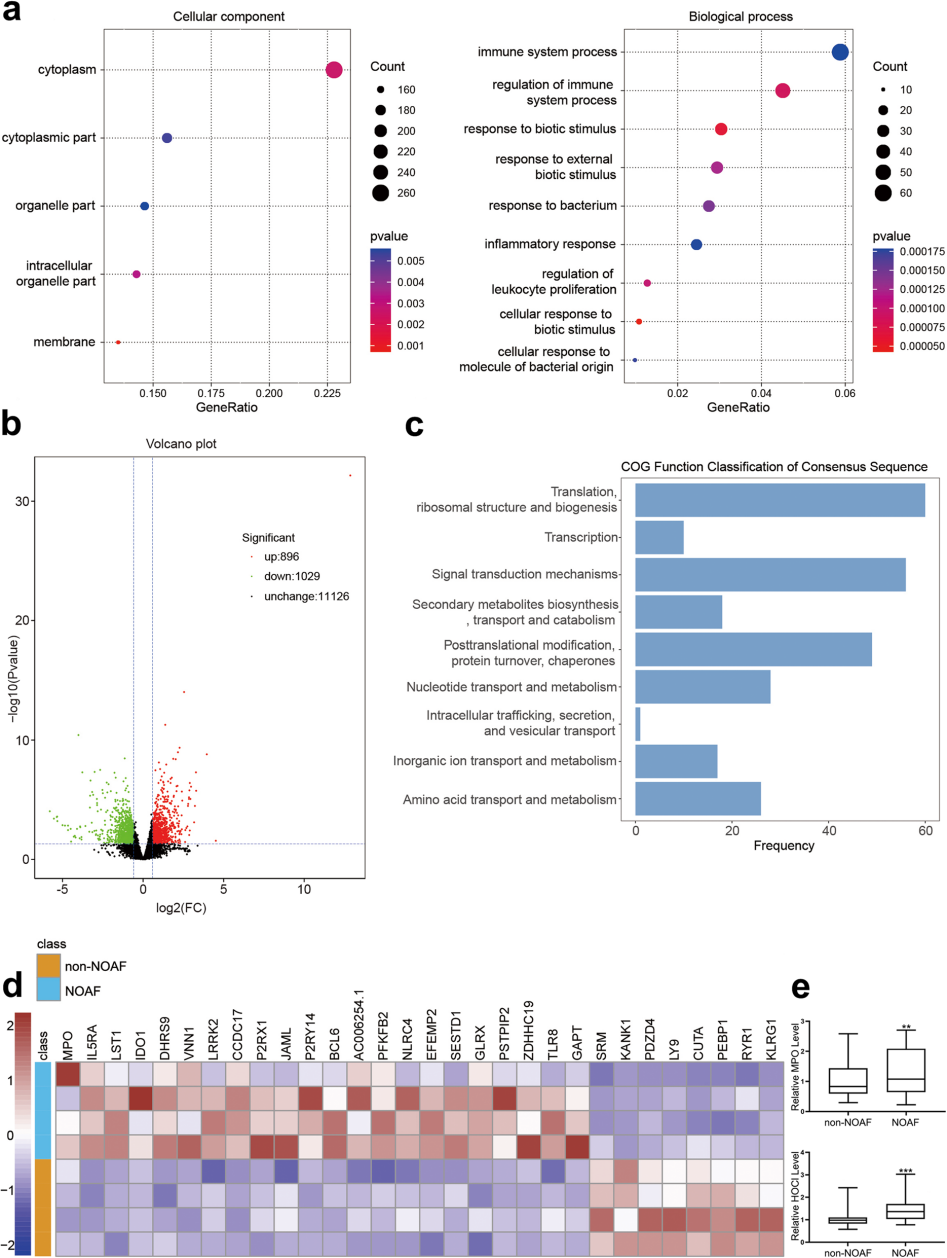
Serum sequencing analysis and determination of MPO and HOCl levels. GO analysis (**a**), volcano map of different genes (**b**), COG analysis (**c**), partial different genes expression (**d**) and MPO and HOCl levels (**e**) in NOAF and non-NOAF group. ** *P*  < 0.01, *** *P*  < 0.001 vs. non-NOAF. COG: cluster of orthologous groups; MPO: myeloperoxidase; NOAF: new-onset atrial fibrillation

To further explore the levels of inflammation in the two groups of patients, deep sequencing analysis was performed on the serum of NOAF and non-NOAF patients. As shown in Fig. 2b, a total of 896 upregulated genes and 1,029 downregulated genes were detected in NOAF patients compared to non-NOAF patients. Enrichment analysis based on gene ontology (GO) revealed differences in biological processes and cellular components (Fig. 2a). Cluster of orthologous groups (COG) analysis also showed several gene function changes in both groups (Fig. 2c). Finally, partial differential gene expression in NOAF and non-NOAF groups was displayed in a heatmap (Fig. 2d). Interestingly, the expression of MPO was significantly increased in the NOAF group. MPO and HOCl levels were further measured by colorimetry and fluorescence colorimetry. As shown in Fig. 2e, the concentration of MPO and HOCl was higher in the NOAF group compared to the non-NOAF group. These results suggest that MPO and HOCl may be predictors of NOAF in sepsis.

### Selection of risk factors for NOAF in sepsis and the construction of a predictive model

**Fig. 3:**
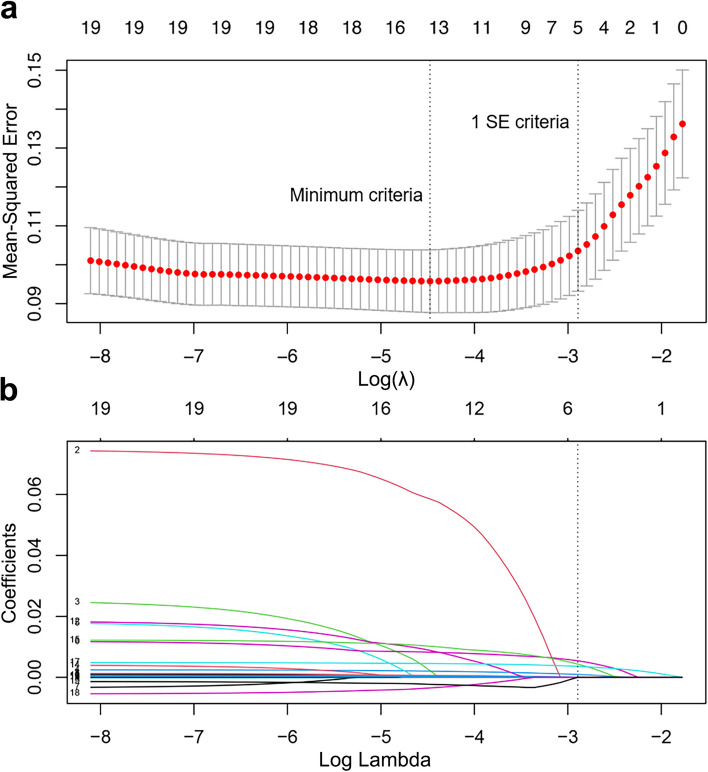
Variable selection using LASSO regression. The tuning parameter (λ) in the LASSO model was selected by 10-fold cross-validation (**a**). The vertical dashed lines represent the best values using the minimum criteria and one standard error of the minimum criteria (1 SE criteria). The λ value of 0.055, with log (λ), -2.893 was selected (1 SE criteria) according to 10-fold cross-validation. LASSO coefficient curves for 19 variables (**b**). A coefficient profile plot was produced for the log(λ) series. A vertical dashed line was drawn on the values chosen by the 10-fold cross-validation method, where the best λ leads to 5 non-zero coefficients. SE: standard error

**Fig. 4:**
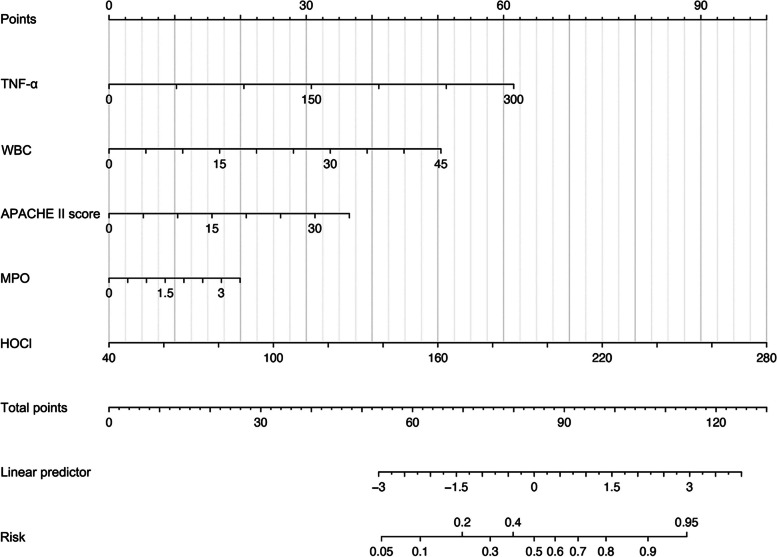
Nomogram model for predicting the risk of NOAF in septic patients. APACHE II: Acute Physiology and Chronic Health Evaluation II; HOCl: hypochlorous acid; MPO: myeloperoxidase; TNF-α: tumor necrosis factor-α; WBC: white blood cells

To better predict the occurrence of NOAF in sepsis, we constructed a predictive model using LASSO and multivariate logistic regression analyses. Based on the data, the important variables associated with the occurrence of NOAF were selected: age, CAD, heart failure, WBC, neutrophils, neutrophil percentage, CRP, PCT, TNF-α, MPO, HOCl, CR, TNI, NT-proBNP, LDL-c, SOFA score, APACHE II score, LVEF, and LVFS. These variables were screened by LASSO regression, and 5 predictors with non-zero coefficients were selected, including HOCl, TNF-α, WBC, APACHE II score, and PCT (Fig. 3). Based on the results of patient serum sequencing and testing, MPO was included for multivariate logistic regression analysis. Finally, after multivariate logistic regression analysis, MPO, HOCl, TNF-α, WBC, and APACHE II score were identified as independent risk factors for NOAF in sepsis (Table 2). We weighted the regression coefficients of the risk factors in multivariate logistic regression and developed a risk score formula for predicting NOAF in sepsis. Risk score = − 9.83 + 0.56 (MPO) + 0.04 (HOCl) + 0.02 (TNF-α) + 0.11 (WBC) + 0.10 (APACHE II score). Predicted risk = 1 / (1 + e^−risk score^). The nomogram model for predicting the probability of NOAF in sepsis was developed based on these risk factors (Fig. 4).

**Table 2:** Relationship between risk factors and NOAF in septic patients

Variables	β	OR (95% CI)	*P* value
Intercept	-9.83		< 0.001
MPO	0.56	1.74 (1.08–2.83)	0.024
HOCl	0.04	1.04 (1.03–1.05)	< 0.001
TNF-α	0.02	1.02 (1.01–1.03)	0.001
WBC	0.11	1.12 (1.06–1.18)	< 0.001
APACHE II score	0.10	1.11 (1.04–1.18)	0.003

*APACHE II *Acute Physiology and Chronic Health Evaluation II, *CI *confidence interval, *HOCl *hypochlorous acid, *MPO *myeloperoxidase, *OR *odds ratio, *TNF-α *tumor necrosis factor-α, *WBC *white blood cells

### Predictive value of independent risk factors for NOAF in sepsis

**Fig. 5:**
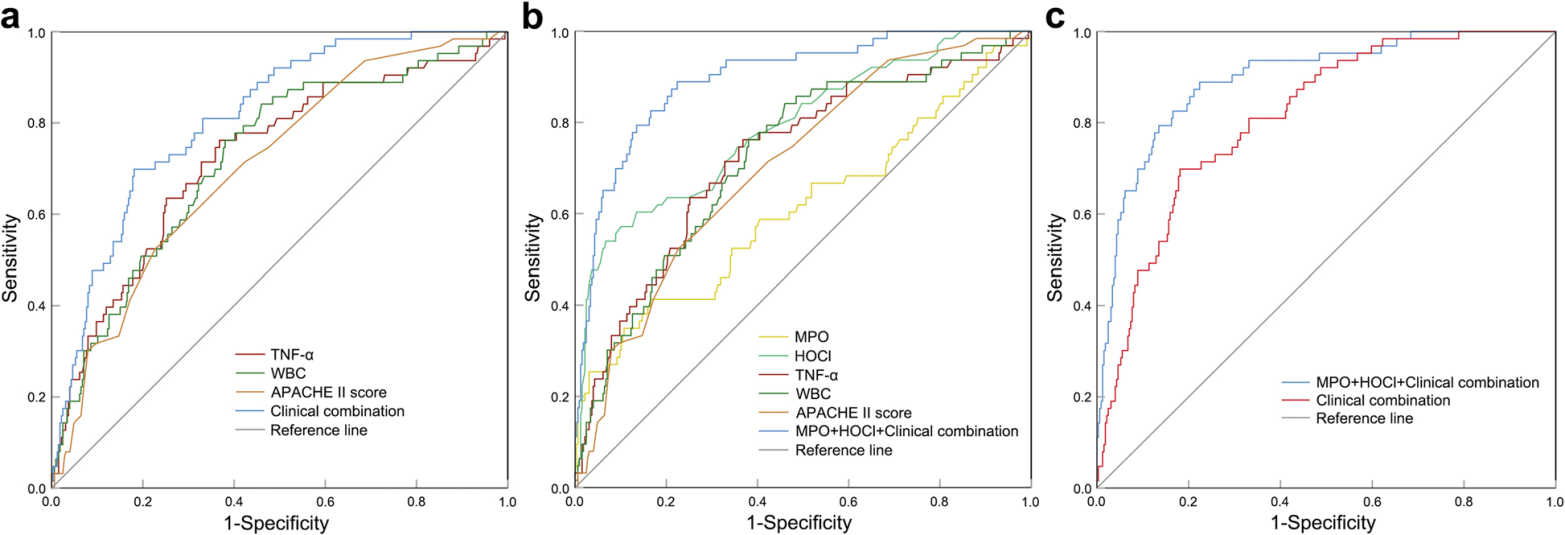
ROC curves for independent risk variables predicting NOAF in sepsis. ROC curves for single and combined TNF-α, WBC, and APACHE II score (**a**). ROC curves for single and combined MPO, HOCl, TNF-α, WBC, and APACHE II score (**b**). Comparison of ROC curves for the two different combined modalities (**c**). *** *P*  < 0.001 vs. AUC of combined TNF-α, WBC, and APACHE II score. Note: Clinical combination: TNF-α + WBC + APACHE II score. APACHE II: Acute Physiology and Chronic Health Evaluation II; HOCl: hypochlorous acid; MPO: myeloperoxidase; TNF-α: tumor necrosis factor-α; WBC: white blood cells

Finally, we analyzed the predictive value of each factor and their combination for NOAF in sepsis. Among individual indicators, HOCl had the strongest predictive efficacy with an AUC of 0.789, followed by TNF-α, WBC, APACHE II score, and MPO with AUCs of 0.724, 0.724, 0.706, and 0.61, respectively. The AUC increased to 0.813 when combining TNF-α, WBC, and APACHE II score (Fig. 5a). The AUC further increased to 0.897 when combining MPO, HOCl, TNF-α, WBC, and APACHE II score (Fig. 5b), which significantly elevated the AUC (*P* < 0.001) (Fig. 5c). These results suggest that the combination of MPO and HOCl with TNF-α, WBC, and APACHE II score could be more effective in predicting the occurrence of NOAF in sepsis.

## Discussion

In our study, a total of 389 patients with sepsis were included, 63 of whom presented with NOAF, resulting in an incidence of 16.20%. Septic patients with NOAF had higher levels of MPO and HOCl compared to those without NOAF. Moreover, we found that MPO, HOCl, TNF-α, WBC, and APACHE II score were independent risk factors for NOAF through multivariate logistic regression analysis. Based on these predictors, we created a nomogram model. Additionally, we analyzed the predictive efficacy of these predictors and found that the combination of MPO and HOCl with TNF-α, WBC, and APACHE II score could better predict the occurrence of NOAF in sepsis.

Risk factors for NOAF in sepsis include advanced age, male sex, respiratory and cardiovascular diseases, and heart and respiratory failure. Many epidemiological studies have shown that advanced age is an independent risk factor for NOAF in patients with sepsis [21–23]. It is well known that advanced age is a risk factor for AF in both in the general population and intensive care unit patients [24]. Myocardial anatomy and electrophysiology change with age. The mean age of patients with NOAF in sepsis in this study was 76 years, but age was not an independent risk factor. Recently, data from an international multicenter (45 countries) CLARIFY registry study showed that NOAF is common in patients with chronic coronary syndromes and is strongly associated with worse outcomes [25]. There may be a bidirectional interaction between the pathophysiology of AF and CAD. On the one hand, AF may be involved in the progression of CAD by exacerbating endothelial dysfunction and systemic inflammation. On the other hand, the presence of CAD may lead to atrial ischemia or infarction, which may contribute to the development of AF through a variety of mechanisms (re-entry phenomena, focal ectopic activity, and autonomic imbalance in favor of the sympathetic system) [26]. Interestingly, the majority of extensively studied AF risk factors to date (including aging, male sex, hypertension, valvular heart disease, left-ventricular dysfunction, obesity, alcohol consumption, smoking, DM, and obstructive sleep apnea) overlap with CAD risk factors [27]. Moreover, CAD and AF share common pathophysiological bases, with inflammation playing a central role in the development and propagation of both AF and CAD [28]. In our study, patients with NOAF had a higher incidence of CAD compared to those without NOAF. However, CAD was not an independent risk factor which may be related to the weaker effect of CAD on NOAF in sepsis.

T lymphocytes are closely associated with the onset and development of various cardiovascular diseases. Helper T lymphocytes and cytotoxic T lymphocytes promote the development and progression of atherosclerosis [29, 30]. The expression of CD3 + lymphocytes in the left atrial appendage is significantly higher in patients with paroxysmal AF and persistent AF than in patients with sinus rhythm [7]. In our study, there was no significant difference in the proportion of T lymphocytes in the peripheral blood between septic patients with and without NOAF. This suggests that NOAF in septic patients may be caused by elevated levels of pro-inflammatory cytokines during sepsis [31].

Previous studies have found that inflammation plays a crucial role in the development of AF [32, 33]. In our study, we observed that inflammatory markers such as WBC, TNF-α, and CRP were significantly elevated in septic patients with NOAF. WBC, an indicator of systemic inflammation, is associated with an increased risk of cardiovascular disease [34, 35]. A higher WBC is positively correlated with a higher risk of AF [36, 37]. In this study, the WBC was significantly higher in the NOAF group compared to the non-NOAF group (median: 14.40 vs. 9.10, *P* < 0.001), and it also served as an independent risk factor for NOAF [odds ratio (OR): 1.12, 95% confidence interval (CI): 1.06–1.18)]. TNF-α is a glycoprotein and a peptide hormone synthesized primarily by monocytes and macrophages. It has been extensively studied in various cardiovascular disease settings [38]. TNF-α stimulates acute immune cell responses and induces inflammation. Serum TNF-α levels are significantly higher in patients with AF compared to those in sinus rhythm and levels are higher in those with persistent and permanent AF than in patients with paroxysmal AF [39]. High serum TNF-α levels are associated with a higher risk of AF. Serum levels at hospital admission in patients with chronic AF also predict the risk of future stroke [40]. In this study, TNF-α was identified as a significant risk factor for NOAF with an OR value of 1.02 (95% CI: 1.01–1.03). Elevated CRP level may be an independent predictor of all-cause mortality, stroke, and major adverse cardiovascular events in patients with AF, and baseline CRP levels can provide important prognostic information for risk classification of patients in AF [41]. A meta-analysis from 2022 including 21 studies concluded that high-sensitivity CRP (hsCRP) is a predictor for AF recurrence after AF-Ablation [42]. In this study, hsCRP data were not available, and CRP levels were higher in the NOAF group than in the non-NOAF group, but CRP was not an independent risk factor.

Inflammatory cell infiltration in the myocardium is associated with an increased risk of AF [43]. Inflammatory indicators can reduce myocardial contractility by upregulating myocardial nitric oxide synthase and downregulating sarcoplasmic reticulum Ca^2+^-ATPase (SERCA) [44]. This infiltration leads to myocardial micro-abscesses and promotes myocardial fibrosis [45]. Our unpublished studies have shown that during sepsis, neutrophils infiltrate the atrium and secrete more MPO. Its derivative HOCl leads to cellular calcium overload by downregulating SERCA expression and activity in atrial myocytes, ultimately contributing to the occurrence of AF. Through deep sequencing analysis of peripheral blood from NOAF and non-NOAF patients, we found that the expression of the MPO gene was significantly increased in the NOAF group. We also measured the levels of MPO and HOCl in the serum by colorimetry and fluorescence colorimetry, finding that their levels were higher in the NOAF group. Further analysis identified both as independent risk factors for NOAF in sepsis (MPO: OR: 1.74, 95% CI: 1.08–2.83; HOCl: OR: 1.04, 95% CI: 1.03–1.05).

The APACHE II score is currently the most widely used clinical score to evaluate the physiological and pathological status and severity of a patient’s condition [46]. Within the first 24 h of a patient’s admission, the worst value of each physiological variable is calculated as an integer score from 0 to 71. Higher scores indicate more severe disease and a higher risk of death in the hospital. Our study suggested that the APACHE II score is an independent risk factor for NOAF in sepsis (OR: 1.11, 95% CI: 1.04–1.18).

Previous studies have also attempted to predict the occurrence of NOAF in sepsis. Wetterslev M systematically analyzed and discussed the risk factors for NOAF in critically ill adult patients but did not develop a simple and practical predictive model [31]. Furthermore, one study developed a risk factor scoring system for NOAF in sepsis, but it was more complex to use and had a C-statistic of 0.81 [21]. A recent study that included 2,492 patients with sepsis showed that age, fibrinogen, CRP, SOFA score, congestive heart failure, and dobutamine use were used as risk variables to create a nomogram model, achieving an AUC of 0.861 [47]. In this study, we constructed a predictive model for NOAF in sepsis using MPO and HOCl combined with TNF-α, WBC, and APACHE II score, which demonstrated a better predictive value with an AUC of 0.897.

This study has some limitations. First, the sample size was small, which may introduce potential comparison bias. Second, morphology-voltage-P-wave duration ECG score and P-wave peak time are important predictors of the risk of developing atrial arrhythmia in a variety of diseases [48–50], but it was not included in our study. In future studies, we aim to expand the sample size and include more indicators to achieve better predictive power for NOAF in sepsis.

## Conclusion

In this study, we found that septic patients with NOAF had higher levels of MPO and HOCl compared to those without NOAF. We developed a nomogram model to predict the incidence of NOAF during sepsis, incorporating MPO, HOCl, TNF-α, WBC, and APACHE II score. This model enables individualized prediction of NOAF in patients with sepsis and offers the possibility of early intervention and prevalence reduction.

## Data Availability

The mRNA sequence data generated and/or analysed during the current study are available in the NCBI under SRA repository, https://www.ncbi.nlm.nih.gov/sra/PRJNA953162. The other data used to support the findings of this study are available from the corresponding author on reasonable request.

## Abbreviations

AF: Atrial fibrillation
ALT: Alanine aminotransferase
APACHE II: Acute Physiology and Chronic Health Evaluation II
AST: Aspartate aminotransferase
AUC: Area under the curve
CAD: Coronary artery disease
CD: Cluster of differentiation
CI: Confidence interval
COG: Cluster of orthologous groups
COPD: Chronic obstructive pulmonary disease
CR: Creatinine
CRP: C-reactive protein
DM: Diabetes mellitus
ECG: Electrocardiogram
ESICM: European Society of Intensive Care Medicine
GO: Gene ontology
HDL-c: High-density lipoprotein cholesterol
HOCl: Hypochlorous acid
hsCRP: high-sensitivity CRP
IL: Interleukin
LAD: Left atrium diameter
LASSO: Least absolute shrinkage and selection operator
LDL-c: Low-density lipoprotein cholesterol
LVEDD: Left ventricular end-diastolic dimension
LVEF: Left ventricular ejection fraction
LVESD: Left ventricular end-systolic dimension
LVFS: Left ventricular fractional shortening
MPO: Myeloperoxidase
NOAF: New-onset atrial fibrillation
NT-proBNP: N-terminal pro-brain natriuretic peptide
OR: Odds ratio
PCT: Procalcitonin
SCCM: Society of Critical Care Medicine
SERCA: Sarcoplasmic reticulum Ca^2+^-ATPase
SOFA: Sequential Organ Failure Assessment
TC: Total cholesterol
TG: Triglyceride
TNF-α: Tumor necrosis factor-α
TNI: Troponin I
WBC: White blood cells
